# Pregabalin for the treatment of generalized anxiety disorder

**DOI:** 10.1192/j.eurpsy.2022.1011

**Published:** 2022-09-01

**Authors:** A. Khallouk, A. Rhaouti, A. Boukdir, S. Stati, H. Nafiaa, A. Ouanass

**Affiliations:** 1 University Psychiatric Hospital Ar-razi, university hospital center, SALE, Morocco, University Of Mohammed V, Rabat,, salé, Morocco; 2 hopital arrazi de sale, Psychiatrie, sale, Morocco; 3 university hospital center, University Psychiatric Hospital Ar-razi, SALE, Morocco; 4 Hôpital psychiatrique ARRAZI de Salé, Psychiatrie, Salé, Morocco

**Keywords:** disorder, Treatment, Anxiety, pregabalin

## Abstract

**Introduction:**

Pregabalin is a treatement with a complexe mechanism of action. It’s an antiepileptic drug used as adjunctive treatment of partial epilepsy, it is also taken in the treatment of neuropathic pain, and generalized anxiety disorder, in addition to epilepsy. Some of the advantages of pregabalin include. pharmacokinetics, safety, and tolerability. Clinical trials have demonstrated the efficacy of pregabalin comparable to benzodiazepines, without risk of abuse.

**Objectives:**

to assess the efficacy of pregabalin in patients with generalized anxiety disorder in the Ar-Razi university psychiatric hospital in Salé in Morocco

**Methods:**

To assess the place of pregabalin in the treatement of anxiety disorders through patients hospitalized in the Ar-Razi university psychiatric hospital in Salé in Morocco The evaluation instruments are: For anxiety the Hamilton Anxiety Scale and For therapeutic efficacy CGI-therapeutic index

**Results:**

based on the results of our study on the patients who have improved after an optimal duration of treatment, in conjunction with psychological monitoring, we retain that pregabalin can significantly improve the quality of life of anxious patients and also guarantee them a better prognosis

**Conclusions:**

Pregabalin was significantly more efficacious for the treatment of psychic and somatic symptoms of generalized anxiety disorder and was well tolerated by most study patients.

**Disclosure:**

No significant relationships.

